# Research on the development trend, evolution, and spatial local characteristics of the intelligent smart medical industry in the Yangtze River Economic Belt

**DOI:** 10.3389/fpubh.2022.1022547

**Published:** 2023-01-12

**Authors:** Xuesen Zhang, Li Yang, Xiaoheng Zhang, Jicheng Xu

**Affiliations:** School of Economics and Management, Anhui University of Science and Technology, Huainan, China

**Keywords:** Yangtze River Economic Belt, smart medical industry, trend evolution, spatial disequilibrium, kernel density estimation, exploratory spatial data analysis

## Abstract

A comprehensive survey of the development trends, trend evolution, and spatial non-equilibrium characteristics of the intelligent smart medical industry in the Yangtze River Economic Belt could provide significant policy implications for optimizing the spatial layout of the integrated development of the smart medical industry in this region. Using the Criteria Importance Though Intercriteria Correlation objective evaluation method for a study period from 2016 to 2020, 11 provinces and cities along the Yangtze River Economic Belt were quantitatively evaluated in relation to the development of the smart medical industry. Accordingly, the application of exploratory spatial data analysis, the kernel density estimation, and the Dagum Gini coefficient and its decomposition method were used to comprehensively evaluate the trends in the Yangtze River Economic Belt's smart medical industry regarding trend evolution and unbalanced spatial characteristics. The overall level of development of the smart medical industry in the Yangtze River Economic Belt was not good. It showed an increasing spatial pattern from the western inland to eastern coastal regions. The development of the artificial intelligence industry in the Yangtze River Economic Belt showed a positive spatial autocorrelation with significant “spatial spillover effects.” The local agglomeration mode was mainly high (a high cluster). In addition, industrial development showed a multi-polarization trend. Although the degree of spatial disequilibrium in the artificial intelligence industry development along the Yangtze River Economic Belt has decreased in recent years, the degree of spatial disequilibrium remains significant.

## Introduction

With the rise of emerging technologies, such as big data, cloud computing, and the Internet of Things, the development of artificial intelligence (AI) has become an inevitable trend and has penetrated all fields of social production (1). In 2017, China released the “New Generation of Artificial Intelligence Development Plan,” and AI development was elevated to the level of a national strategy (2, 3). According to the relevant guidelines in the report from the 19th National Congress of the Communist Party of China, China's next development direction will be to improve the development efficiency of the manufacturing industry, promote the integration of AI and the real economy, and build manufacturing power. AI has also become an indispensable driving force for transforming Chinese enterprises to develop precision, quality, and efficiency (4). Based on the urban agglomeration development planning promulgated in 2016 in the Yangtze River Delta, the old and obsolete kinetic energy of traditional enterprises has been transformed, and the traditional economic development orientation and paths have been innovated, which is included in the current industrial reform project. As an example of industrial optimization based on artificial intelligence in the traditional medical industry, the smart medical industry is the carrier and manifestation of breaking with the old and establishing the new (5). Therefore, artificial intelligence has become an important technological path to facilitate the transformation of the healthcare industry and achieve new economic growth in the Economic Belt of the Yangtze River.

Recently, research on smart medicine has mainly focused on policy and development suggestions. For example, Shi-Kui et al. (6) argued that the current large market demand in China is the basis for the development of smart medicine; thus, various institutions and research organizations should continuously improve their investments in smart medicine, and there are great prospects and space for the smart medical industry's development. According to Xiaoyu et al. (7), health management, intelligence level, and clinical decision-making are important manifestations of the combination of AI and the healthcare industry. However, few quantitative studies have been conducted on the development of the current state of the smart healthcare industry. From an industrial efficiency development perspective, Zhixian (8) introduced a data quality assessment algorithm to study the efficiency of smart medical modernization. According to Wang et al. (9), it is difficult to maintain the integrity of basic data on smart medical care, the field of smart medical technology lacks innovative algorithms, and application-oriented products are difficult to implement.

In summary, qualitative research in the smart healthcare industry provides theoretical support for further dynamic quantitative research, and there remains much room for current research. First, the evaluation of existing indicators for the development characteristics of the smart medical industry is affected by factors such as sample size and calculation models and typically uses a single indicator, which cannot ensure the accuracy and comprehensiveness of the research results. Second, current research on the smart medical industry focuses on evaluating provincial and municipal units, with little research conducted on the Yangtze River Economic Zone. Third, although scholars have recently explored the spatial differences of specific regions in relation to the spatial agglomeration of the smart medical industry, few studies have been conducted on the reasons for the differences and unbalanced characteristics in the spatial distribution of the smart medical industry, and policy recommendations for the integrated development of the smart medical industry in the Yangtze River Economic Belt are insufficient.

Criterion importance through intercriteria correlation (CRITIC) is a method for assigning weights to indicators by considering the difference information between evaluation indicators and the similarity information between two indicators, which assigns smaller weights to indicators with a large horizontal similarity and larger weights to indicators with a large vertical difference. Finally, the horizontal similarity and vertical difference information are multiplied to assign weights to the indicators (10). In this study, we borrowed the idea of the CRITIC method to determine the combination weights in the combination evaluation method. We first determined the vertical differences and horizontal similarities of different evaluation methods separately and then combined the horizontal similarity and vertical difference information to obtain the combination weights. The basic idea is to establish the objective weights of indicators based on the discriminative power and conflict between evaluation indicators. The discriminative power of an indicator refers to the variability of the same indicator across different samples. The greater the variability of an evaluation indicator in different samples, the stronger its discriminative power; on the contrary, the smaller the variability of an evaluation indicator in different samples, the weaker its discriminative power. The standard deviation is used to measure the discriminative power of indicators (11); the conflict of indicators refers to the correlation between indicators and the correlation coefficient and is used to measure the size and direction of the conflict. In summary, the discriminative power and conflict between indicators are used to measure the weight of each indicator, which is the basic idea of the CRITIC method.

Exploratory spatial data analysis is essentially a “data-driven” analysis method that analyzes and identifies the properties of spatial information to guide the structure of and solution for deterministic models. The ESDA methods can be divided into global statistics and local statistics. Global statistics mainly explore the distribution characteristics of an attribute in a region. By analyzing sub-regions' information separately, local statistics can explore whether the changes in regional information are smooth (homogeneous) or there are mutations (heterogeneous). The concept of spatial autocorrelation was first proposed by Cliff and Ord (12) and then cited and revised by many scholars (13). Based on this, spatial statistical methods of spatial dependence and heterogeneity were proposed, revealing the spatial correlation relationship of geographical phenomena in geographical distribution (14, 15). Moran's scatterplot studies local spatial instability by visualizing a two-dimensional representation of Wz and z data pairs. The Moran scatter plot is marked in the coordinate system, with the deviation z of the observed values in each region as the abscess and the spatial lag value Wz as the ordinate. The four different quadrants correspond to four different local spatial connections between a region and its neighbors. The regions in the first quadrant on the upper right of the Moran scatter plot have large observed values, and the nearby regions also have large observed values. Such regions are called “high-high” (HH) type regions. Areas in the second quadrant on the upper left of the Moran scatter plot have smaller observed values, but nearby areas have larger observed values. Such areas are called low-high (LH) areas. In this way, the areas located in the lower left of the third quadrant of the Moran scatter plot are called low-low (LL) type areas, and the areas located in the fourth quadrant at the bottom right the Moran scatter plot are called low-high (LH) areas. The first (HH) and third (LL) quadrants indicate positive spatial autocorrelation, indicating the spatial clustering of similar observations. In contrast, the second (HL) and fourth (LH) quadrants indicate negative spatial autocorrelation, indicating the spatial clustering of different observations. Therefore, this study investigated the unbalanced characteristics of the spatial development of the smart medical industry and its development pattern in the economic belt and cities. We first adopted the bowstring and arrow model to summarize the development evaluation index of the smart medical industry in the Yangtze River economic zone, then summarized the development trends and current characteristics of the smart medical industry in the Yangtze River Economic Zone, and finally proposed targeted policy recommendations. This study not only enriches the current theoretical research on the smart medical industry in the Yangtze River Economic Zone but also provides a basis for decision-making on the coordinated and integrated development of the smart medical industry in the region.

## Indicator system

The bowstring arrow theoretical model is applied to evaluate the competitiveness of cities and industrial clusters. The index system is divided into two parts, in which “hard power” refers to the material resources a city possesses, including eight indicators (e.g., labor force, capital market, land, technology). Soft power refers to a city's flexible resources, including five indicators (e.g., a city's culture, openness, and management power).

According to the outline of the Yangtze River Economic Belt Development Plan, the Yangtze River contains nine provinces and two cities. The upper, middle, and lower reaches of the Yangtze River are divided as follows: Heyuan—Yichang in Hubei is the upper reaches, including Sichuan, Yunnan, Chongqing, and Guizhou; Yichang in Hubei—Hukou in Jiangxi is the middle reaches, including Hubei, Hunan, and Jiangxi; and below the mouth of Jiangxi Lake are the lower reaches, including Shanghai, Jiangsu, Zhejiang, and Anhui. In 2018, China released the Classification of Emerging Strategic Industries (16, 17). Table 1 shows the classification of the smart medical industry together with the corresponding relationships with national economic industries.

**Table 1:** Classification of the smart medical industry in strategic emerging industries (2018).

**Industry name**	**Industry classification name**	**National economy industry code**	**National economy industry name**
Smart medical industry	Smart medical software development	6511*	Basic software development
		6513*	Application software development
	Smart medical-related equipment manufacturing	3961*	Wearable smart device manufacturing
		3963*	Smart medical device manufacturing
		3969*	Other smart medical consumer equipment manufacturing
		3990*	Other electronic equipment manufacturing
	Smart medical system services	6531*	Information system integration services

Both the classification in Table 1, the national economic industries corresponding to the software and information technology service industries in “the High Technology Industry (Service Industry) Classification (2018)” and “High Technology Industry (Manufacturing Industry) Classification (2018),” and the national economic industries corresponding to the development of smart medical software and smart medical system services in Table 2 are consistent with each other; thus, the data are mainly from the “China Information Industry Yearbook” and “China Electronic Information Industry Yearbook.” Therefore, in this study, to more accurately calculate the results of the development of the smart medical industry, the above-mentioned basic industries mentioned above and the smart consumption-related equipment manufacturing industry were grouped into the electronic and communication equipment manufacturing industry, for which data were mainly obtained from the “China High Technology Industry Statistical Yearbook.”

**Table 2:** Evaluation index system of smart medical industry development in the Yangtze River Economic Zone.

**Guideline layer**	**Primary indicators**	**Secondary indicators**	**Data source**
Hard force	Workforce	Average number of employees in the smart medical industry	China Information Industry Yearbook, China High-Tech Industry Statistical Yearbook
		Number of R&D personnel in the smart medical industry	China Information Industry Yearbook, China High-Tech Industry Statistical Yearbook
		Average number of students in colleges and universities per 100,000 population	China Torch Statistical Yearbook, China High-Tech Industry Statistical Yearbook
		Education expenses	China Statistical Yearbook
	Capital power	Total assets of the smart medical industry	China Statistical Yearbook
		Total profit of the smart medical industry	China Information Industry Yearbook, China High Technology Industry Statistical Yearbook
		Smart medical industry's main business income	China Information Industry Yearbook, China High Technology Industry Statistical Yearbook
		Total fixed asset investment in the smart medical industry	China Information Industry Yearbook, China High Technology Industry Statistical Yearbook
	Technology power	Number of smart medical patents granted	Statistical Yearbook of Fixed Asset Investment in China
		Number of research institutions in the smart medical industry	Statistical Yearbook of Science and Technology Activities of Industrial Enterprises
		Total R&D expenditure for regional industries	Statistical Yearbook of Science and Technology Activities of Industrial Enterprises
		Total R&D funding for the smart medical industry	China Industrial Statistical Yearbook
		R&D investment intensity of the smart medical industry	Statistical Yearbook of Scientific and Technological Activities of Industrial Enterprises
	Structural forces	Proportion of sales revenue of new products in high technology industry	Statistical Yearbook of Scientific and Technological Activities of Industrial Enterprises
		Smart medical industry concentration	China Statistical Yearbook, China Electronic Information Industry Yearbook
		Contribution of smart medical industry to regional GDP	China Industrial Statistical Yearbook, China Information Industry Yearbook
		GDP per capita	China Information Industry Yearbook China Statistical Yearbook
		Per capita consumption expenditure of all residents	China Statistical Yearbook
		Smart medical enterprise size structure	China Statistical Yearbook
		Number of people with Internet access	Statistical Yearbook of Scientific and Technological Activities of Industrial Enterprises, China Statistical Yearbook
		Internet penetration rate	China Statistical Yearbook
	Facility power	Grade road mileage	China Statistical Yearbook
		Total cargo volume	Statistical Yearbook of Provinces and Cities
		Total passenger traffic	Statistical Yearbook of Provinces and Cities
	Location power	Regional GDP as a percentage of national GDP	Statistical Yearbook of Provinces and Cities
	Environmental forces	Total investment in environmental pollution control	China Statistical Yearbook
		Total investment in environmental pollution control as a percentage of GDP	Statistical Yearbook of Provinces and Cities
		China Statistical Yearbook Green Covered Area	China Statistical Yearbook
	Gathering power	Total city population	China Statistical Yearbook
		Urban population growth	China Statistical Yearbook
		Number of smart medical companies	China Statistical Yearbook
Soft force	Power of order	Price index growth rate	Statistical Yearbook of Scientific and Technological Activities of Industrial Enterprises
		Variance of GDP growth rate in the last 5 years	Statistical Yearbook of Provinces and Cities
	Cultural power	Number of R&D activities in artificial intelligence companies as a percentage of companies	Statistical Yearbook of Provinces and Cities
		Number of colleges and universities that offer artificial intelligence majors as a percentage	Statistical Yearbook of Scientific and Technological Activities of Industrial Enterprises
		Number of artificial intelligence enterprises with research institutions as a percentage	China Statistical Yearbook "China Electronic Information Industry Yearbook
		Percentage of new product sales companies among artificial intelligence companies	Statistical Yearbook of Scientific and Technological Activities of Industrial Enterprises
	System power	Regional tax revenue as a share of fiscal revenue	Statistical Yearbook of Scientific and Technological Activities of Industrial Enterprises
		Percentage of regional agency staff	China Statistical Yearbook
	Management power	Number of management staff	Statistical Yearbook of Provinces and Cities
		Percentage of those engaged in the AI industry with a master's degree and above	Statistical Yearbook of Provinces and Cities
	Open power	Total exports of high technology products	China Statistical Yearbook China Electronic Information Industry Yearbook
		Total imports of high-tech products	China Trade and Foreign Economic Statistical Yearbook
		Number of overnight visitor arrivals from abroad	China Trade and Foreign Economic Statistics Yearbook
		Export value of high-tech products as a percentage of regional GDP	China Trade and Foreign Economic Statistics Yearbook

The secondary index was obtained by extracting the high-frequency vocabulary from the goals and requirements of smart medical industry development.

Based on the analysis of the above bowstring arrow theoretical model, combined with the objectives and requirements for the development of the smart medical industry in the above Action Plan, New Generation Artificial Intelligence Development Plan, and Yangtze River Delta City Cluster Development Plan (18), the basic principles of systematicity, science, operability, and objectivity were followed in the construction of the evaluation index system. This study, based on the two guideline levels of hard and soft forces, used the bowstring arrow theoretical model as the basis for constructing evaluation indices for the smart medical industry in the Yangtze River Economic Zone, including 13 primary indicators and 45 secondary indicators, such as labor force, capital force, and technology force (Table 2).

## Research methodology

### CRITIC method

This study used the Criteria Importance Though the Intercriteria Correlation method to assign values to the research indicators by comparing each indicator's strength and conflicting nature, fully considering the relevant indicator data information, and exploring the gap and relevance of each indicator, taking its values and its calculation idea as described below.

First, we determined the research object and evaluation indices, corresponding to the numbers m and n, respectively, to define and assign values to the research object. At this time, the value of the j-th evaluation index of the ith evaluation object is recorded as Xij, constructing all evaluation indices into a matrix. Each index's standard deviation Si and correlation coefficient Pkl can be found in the following ways:


$$\begin{array}{l} S_{\text{i}}=\sqrt{\frac{1}{\text{m}}\sum_{\text{j}=1}^{\text{m}}{\left({\text{x}}_{\text{ij}}\text{−}\bar{{\text{x}}_{\text{ij}}}\right)}^{2}}\\ {\text{ P}}_{\text{kl}}\text{=}\frac{\text{cov}({\text{X}}_{\text{k}}{}^{\text{*}},{\text{X}}_{\text{l}}{}^{\text{*}})}{{\text{S}}_{\text{k}}{\text{S}}_{\text{l}}}, \end{array}$$

where ${\bar{x}}_{ij}$ is the mean of the jth indicator (i.e., the mean of the elements of the ith row of the standardized matrix), and X* denotes the covariance of the kth and Ith rows of the standardized matrix X*. Next, the size of the information represented by each indicator Cj determines the richness of the information contained in the jth indicator and the size of the weight coefficient wj of each indicator, which is calculated as follows:


$$\begin{array}{l} {\text{C}}_{\text{j }}{\text{= s}}_{\text{j}}\;\sum_{\text{l=1 }}^{\text{n }}(\text{1}-{\text{p}}_{\text{kl}}\text{) }\\ {\text{ W}}_{\text{j }}\text{=}\frac{{\text{C}}_{\text{j}}\;}{\sum_{\text{l =1}}^{\text{n }}{\text{C}}_{\text{j}}} \end{array}$$

Finally, based on the above weighting coefficients, the development of the evaluation value of the smart medical industry in each region for each year was calculated using the linear weighting method and recorded as follows:


$${\text{Z}}_{\text{i}}\text{=}\sum_{\text{j = 1}}^{\text{n}}{\text{w}}_{\text{j}}{\text{x}}_{\text{ij}}$$

### Moran's I exploratory spatial data analysis method

To explore the relationships, patterns, and trends hidden in the data, first, the data's patterns and characteristics were separated to reveal the deviations of the data from common models. Then, the characteristics and patterns of the spatial distribution of smart medical care in the Yangtze River Economic Zone were specifically divided into global and local autocorrelation analyses (19). This study measured the degree of spatial correlation and aggregation of the smart medical industry in the Yangtze River Economic Belt, judged by Moran's I index, with −1 and 1 as the critical values. An index >0 indicates that the spatial distribution between provinces in the Yangtze River Economic Belt has a positive correlation and that development is more aggregated, and an index equal to 0 indicates no correlation. An index <0 indicates that, in the Yangtze River Economic Belt, the spatial distribution between provinces is characterized by a negative correlation relationship that is more discrete. The larger the absolute value of Moran's I, the more obvious the clustering (discrete) characteristic representing the development of the smart medical industry. The formula is as follows:


$$\text{Moran′s I}=\text{m }\sum_{\text{i }=\text{1,j }=\text{1}}^{\text{m }}\frac{{\text{W}}_{\text{ij}}{\text{(Y}}_{\text{i}}-\text{Y})({\text{Y}}_{\text{j}}-\text{Y})}{\sum_{\text{i }=1,\text{j }=1}^{\text{m }}W_{\text{ij}}{{\text{(Y}}_{\text{i}}-\text{Y})}^{\text{2}}\;}$$

### Dagum Gini coefficient and decomposition method

Based on the surveyed provinces, to determine the causes of regional differentiation, this study used the Dagum Gini coefficient and the decomposition method to decompose the overall Gini coefficient G into intra-regional variation contribution Gw, inter-regional net value variation contribution Gnb, and super-variable density contribution Gt; the three satisfying G = Gw + Gnb + Gt (20). The three denote regional differences, development differences, and the degree of crossover of development differences (21).

## Empirical analysis

The data sources used for this study were mainly the China Statistical Yearbook, the Yangtze River Economic Belt Development, the Statistical Yearbook of the Yangtze River Economic Belt, the Statistical Bulletin of the Yangtze River Economic Belt, and the Statistical Yearbooks of provinces and cities in the Yangtze River Economic Belt (22, 23). For the higher-order indicators used in the calculation process (e.g., the R&D input intensity of the smart medical industry, the concentration of the smart medical industry, the contribution of the smart medical industry to regional GDP, and the scale structure of smart medical enterprises), it was necessary to budget and process them in advance to obtain data before beginning the actual calculations. The data were initially processed for consistency and invariance after the higher-order indicators were calculated because the extreme difference processing method has unique advantages compared to several other methods. The extreme difference processing method was used in this study to perform dimensionless processing on each index (24, 25).

### Development of smart medical industry regions in provinces and cities in the Yangtze River Economic Belt

According to the steps of the CRITIC method, the data of each index were substituted into the formula (1–4). The weighted coefficients of each index were measured using MATLAB software and then substituted into formula (5) to obtain the evaluation value and ranking of the development of the smart medical industry in 11 provinces and cities in the economic belt of the Yangtze River from 2016 to 2020 (Table 3). As we can see in the steps above, the evaluation values for the hard and soft strengths of the smart medical industry in each province and city in the Yangtze River Economic Belt from 2016 to 2020 are shown in Table 4.

**Table 3:** Evaluation value and ranking of intelligent medical industry development in the Yangtze River Economic Belt, 2016-2020.

	**2016**		**2017**		**2018**		**2019**		**2020**	
**Region**	**Value of evaluation**	**Ranking**	**Value of evaluation**	**Ranking**	**Value of evaluation**	**Ranking**	**Value of evaluation**	**Ranking**	**Value of evaluation**	**Ranking**
Shanghai	0.4565	3	2.3872	3	0.4354	3	0.4474	3	0.4420	3
Jiangsu	0.7168	1	0.6696	1	0.6923	1	0.6513	1	0.6544	1
Zhejiang	0.4849	2	0.4748	2	0.4879	2	0.4652	2	0.5134	2
Anhui	0.2767	5	0.3131	5	0.3123	4	0.2908	5	0.3088	4
Jiangxi	0.1751	10	0.1609	10	0.1741	10	0.1694	10	0.1900	10
Hubei	0.2573	6	0.3067	6	0.2753	6	0.2823	6	0.3081	6
Hunan	0.2488	7	0.2682	7	0.2746	7	0.2784	7	0.2656	8
Chongqing	0.2183	8	0.2314	8	0.2521	8	0.2446	8	0.2727	7
Sichuan	0.3159	4	0.3491	4	0.3061	5	0.2966	4	0.2987	5
Guizhou	0.1168	11	0.0938	11	0.1088	11	0.1208	11	0.1023	11
Yunnan	0.1808	9	0.2196	9	0.1824	9	0.2098	9	0.2410	9

**Table 4:** Evaluation values of hard and soft sub-power of smart medical industry development in Yangtze River Economic Zone, 2016–2020.

**Region**	**2016**		**2017**		**2018**		**2019**		**2020**	
	**Hard parting force**	**Soft parting force**	**Hard parting force**	**Soft parting force**	**Hard parting force**	**Soft parting force**	**Hard parting force**	**Soft parting force**	**Hard parting force**	**Soft parting force**
Shanghai	0.2766	0.1799	0.2562	0.1710	0.2510	0.1844	0.2370	0.2104	0.2372	0.2048
Jiangsu	0.4878	0.2289	0.4688	0.2008	0.4795	0.2128	0.4660	0.1853	0.4500	0.2044
Zhejiang	0.3296	0.1553	0.3326	0.1423	0.3397	0.1483	0.3376	0.1276	0.3696	0.1438
Anhui	0.1981	0.0787	0.2285	0.0846	0.2294	0.0829	0.2091	0.0816	0.2248	0.0840
Jiangxi	0.1208	0.0543	0.0964	0.0646	0.1098	0.0644	0.1201	0.0493	0.1512	0.0388
Hubei	0.1805	0.0767	0.2048	0.1019	0.1984	0.0769	0.2102	0.0721	0.2400	0.0682
Hunan	0.1816	0.0672	0.1930	0.0753	0.2031	0.0714	0.2133	0.0651	0.1691	0.0966
Chongqing	0.1231	0.0953	0.1247	0.1067	0.1486	0.1034	0.1566	0.0880	0.1690	0.1038
Sichuan	0.1844	0.1314	0.2240	0.1250	0.2047	0.1014	0.1924	0.1043	0.1897	0.1089
Guizhou	0.0457	0.0712	0.0481	0.0457	0.0560	0.0529	0.0651	0.0558	0.0573	0.0450
Yunnan	0.0893	0.0914	0.1012	0.0118	0.0801	0.1023	0.0881	0.1218	0.1025	0.1385

According to the calculation results in Table 3, during 2016–2020, the development of the smart medical industry in the downstream region of the Yangtze River Economic Belt was higher. This was related to the scientific and technological strength, labor force quality, innovation ability, and regionally coordinated development ability of the upstream region. Jiangsu Province performed particularly well for five consecutive years with the first comprehensive industrial evaluation score. Due to its proximity to the sea, Zhejiang Province has been influenced by foreign cultures and has a high degree of cultural openness; investment in smart medical industry technology is high, ranking second and third, respectively. In recent years, Shanghai has experienced rapid economic development, with a number of science and technology-oriented talent introductions; making the development of AI and the information industry strong, making the development of the smart medical industry in 2020 more rapid, and maintaining the comprehensive score to third place. Anhui Province and Sichuan Province have continuously maintained a medium level, but when the two provinces are compared, Anhui Province has outstanding hard strength, while Sichuan Province has soft strength—each has obvious advantages. Hubei, Hunan, and Chongqing provinces have smart medical industries that have been developing in tandem, and the comprehensive score was between six and eight. Although soft power growth was slow, Hunan and Hubei provinces have displayed steady development in hard power in recent years. Jiangxi, Guizhou, and Yunnan are economically underdeveloped owing to their geographical location, lack of talent, and insufficient investment in scientific and technological research and development; thus, the comprehensive score falls at the tail end, and development is slow.

To further explore the spatial distribution pattern of the smart medical industry's development in the Yangtze River Economic Belt, the evaluation values of smart medical industry development in 11 provinces and cities from 2016 to 2020 were divided into four levels from high to low according to Jenks' best natural break method (lower level 0–0.25, moderate level 0.25–0.5, good level 0.5–0.75, better level 0.75–1) (26).

According to the calculation results shown in Tables 3, 4, the smart medical industry in the Yangtze River Economic Zone is currently relatively underdeveloped. The development has remained at the second level and below, with no prominent development provinces. The internal development convergence falls short, largely showing a decreasing trend from developed cities in the east to the west. The smart medical industry is centered around the downstream region, forming an agglomeration of smart medical facilities, especially in Jiangsu and Zhejiang. As Table 3 indicates, Shanghai has developed rapidly in recent years. Although the level is not high, the development rate is indistinguishable from that of Jiangsu and Zhejiang. The smart medical industry is developing faster than other areas of the Yangtze River Delta. This is because the downstream region has a large population of scientific and technological talent, large investments in science and technology, complete information technology and other infrastructure, and significant advantages in developing smart medical technologies. However, Jiangxi, Yunnan, and Guizhou are close to the inland, underdeveloped economically, lack innovation and innovative awareness, and have a poor overall degree of smart medical sector development.

From the dynamic evolution of spatial development patterns, the cities with high development levels are increasing. Table 3 shows the level of development of smart healthcare in each region. Compared to 2016, in 2018, Chongqing increased from the third to the second developmental level. During this period, Zhejiang also changed from a medium to a higher level of development; therefore, it can be seen that the smart medical industry's overall development in the Yangtze River's economic belt is increasing. Further, the infrastructure and related industries along the route are also improving, creating a favorable environment for development and resolving issues with capital investment and technological expertise.

### Spatial aggregation state and evolutionary trend of the smart medical industry in the Yangtze River Economic Zone

To explore the overall spatial correlation characteristics of the smart medical industry in the Yangtze River Economic Belt, we used the global autocorrelation method to analyze the general autocorrelation distribution of observations across space. Using OpenGeoDa software, the global Moran I indices of smart medical industry development in the Economic Zone of the Yangtze River for each year of the study period were calculated as shown in Table 5, where Moran I is the global Moran's index, E (i) is the mathematical expectation, Sd is the standard deviation, Mean is the mean, and P (I) represents the significance level.

**Table 5:** Global Moran's I index for smart medical industry development in the Yangtze River Economic Zone.

**Year**	**Moran's I**	**E (1)**	**Sd**	**Mean**	**P (1)**
2016	0.4368	−0.1	0.1554	−0.0988	0.02
2017	0.3643	−0.1	0.1776	−0.1181	0.02
2018	0.4338	−0.1	0.1806	−0.1003	0.01
2019	0.4418	−0.1	0.1664	−0.1194	0.02
2020	0.4446	−0.1	0.2018	−0.0887	0.02

Table 5 shows the overall development of the smart medical industry in the Economic Belt of the Yangtze River. The Moran I index remains between (0.3643, 0.4446), which is significant at the 5% level, indicating that the smart medical industry shows spatial aggregation. Between 2016 and 2020, Moran's I index was >0, indicating that the development of the smart medical industry in the Yangtze River Economic Zone showed a strong autocorrelation in space. The spatial aggregation of the development of the smart medical industry in the Yangtze River Economic Zone in 2016 and 2017 shows a significant decrease; however, during 2017–2020, the Moran I index continued to increase and showed a gradual trend toward stability, indicating that the spatial autocorrelation trend of the development of the smart medical industry in the Yangtze River Economic Zone continued to climb after 2017. The aggregation trend has tended toward a stable state.

**Figure 1:**
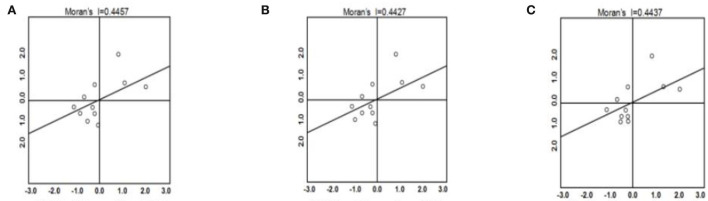
**(A–C)** Moran's I scatter plot for 2016, 2018, and 2020.

OpenGeoDa technology was used to draw a Moran I scatter diagram of the smart medical industry in the Yangtze River Economic Zone in 2016, 2018, and 2020, respectively, with three forms of expression. H-H indicates an area with a high level of development of the smart medical industry and high surrounding development levels; H-L indicates an area that has a high level of smart medical industry development but is surrounded by low development levels, referred to as a “potential area;” and L-L indicates an area with a low level of smart medical industry development that is also surrounded by low development levels, referred to as a “barren area.” As Figure 1 shows, it can be concluded that the Yangtze River Economic Zone experienced H-H performance between 2016 and 2020 in Jiangsu, Zhejiang, and Shanghai, which was closely related to factors such as the construction of the local smart medical infrastructure, the quality of labor, and the early origin of industrial development. The development of the region's own functional medical industry has also driven the rapid development of the smart medical industry in the surrounding areas, forming an area of effect.

Between 2016 and 2020, the L-L and H-L areas were in a state of constant change. Taking Sichuan Province as an example, we can see that it is located in the inner region of the central and western parts of China, and the natural conditions present disadvantages. In the early stage, its use of its smart medical-related industrial base, government policy support, and the introduction of high-quality talent placed the level of development of the smart medical industry always at the forefront in the Sichuan province. However, the surrounding region did not overcome natural conditions, and the development rate was relatively slow, forming the H-L agglomeration. At a later stage, the development of the smart medical industry in Sichuan Province encountered a bottleneck due to factors such as insufficient innovation, weak soft power, and low R&D capability. In 2108, the agglomeration state in Sichuan Province changed from H-L to L-L.

### Uneven development of smart medical industry space in the Yangtze River Economic Zone

The above analysis reveals spatial unevenness in the smart medical industry development of the Yangtze River Economic Zone. Therefore, to further reveal the magnitude of these spatial differences and their sources, the abovementioned Dagum Gini coefficient was used, and its basic decomposition steps by subgroups method were applied. The results of the Dagum Gini coefficient and its decomposition are shown in Table 6.

**Table 6:** Dagum Gini coefficient of smart medical industry development in the Yangtze River Economic Zone and its decomposition.

**Year**	**Overall G**	**Between-group differences**			**Within-group differences**		
		**(2–1)**	**(3–1)**	**(3–2)**	**1**	**2**	**3**
2016	0.2733	0.1614	0.3977	0.3539	0.1869	0.0788	0.1708
2017	0.2554	0.1991	0.3558	0.3090	0.2132	0.1295	0.1452
2018	0.2587	0.1636	0.3806	0.3260	0.1908	0.0912	0.1515
2019	0.2404	0.1514	0.3543	0.3053	0.1580	0.1010	0.1452
2020	0.2368	0.1480	0.3472	0.3005	0.1663	0.1010	0.1415
**Year**	**Overall G**	**Ingredient breakdown**			**Contribution rate**		
		**G**_w_	**G**_nb_	**G**_t_	**G**_w_	**G**_nb_	**G**_t_
2016	0.2733	0.0555	0.1995	0.0183	0.2297	0.8259	0.0759
2017	0.2554	0.0555	0.1758	0.0242	0.2294	0.7278	0.1000
2018	0.2587	0.0523	0.1922	0.0143	0.2166	0.7954	0.0591
2019	0.2404	0.0486	0.1773	0.0145	0.2014	0.7339	0.0600
2020	0.2368	0.1740	0.1740	0.0140	0.2015	0.7205	0.0579

“1” in the table indicates upstream areas, “2” indicates midstream areas, and “3” indicates downstream areas.

#### Analysis of time comparisons of development unevenness within regions

The data indicate that the overall spatial non-equilibrium of smart medical industry development in the Yangtze River Economic Belt is narrowing, while the degree of spatial non-equilibrium and evolutionary trends in each region within the Yangtze River Economic Belt requires further exploration (Table 6). Between 2016 and 2020, the level of internal development of smart medical devices within the upper, middle, and lower reaches of the region varied widely, with a higher level of development in the upper reaches. For example, the Dagum coefficient in the upstream region increased from 0.1869 to 0.2132 in 2016–2017, with a faster growth rate, and then declined in 2 years from 2018 to 2019, reaching the lowest point in 2019. From the overall evolutionary trend, although the changing trend of internal disequilibrium varied between the upstream and downstream regions, the spatial Gini coefficient of each region showed a decreasing trend during the sample study period, and the internal disequilibrium of the development of the smart medical industry decreased.

The reduction of internal non-equilibrium characteristics was mainly caused by the faster development rate of inland city economies, especially in the Sichuan Province region. Economic development has driven the improvement of science and technology levels, the introduction of technical talent, and the construction of smart medical infrastructure, providing the basis for coordinated development among regions. The overall development differences in the Yangtze River Economic Belt decreased, and the close intraregional ties formed a state of mutual economic pull, while the Yangtze River Economic Belt comprehensively promoted the integration of regional science and technology innovation and the integration of mechanical innovation and other aspects of integrated development, prompting the development of each region and further reducing the unevenness of development in each region.

#### Spatial comparison analysis of regional unevenness

Spatial Gini coefficients were obtained among the segments by decomposing the overall Gini coefficient to reveal the degree of disequilibrium between the regions of the upper, middle, and lower reaches of the economic belt of the Yangtze River and their evolutionary trends.

First, from a global perspective, the development of the smart medical industry differed greatly between the upstream and downstream regions of the economic belt of the Yangtze River during 2016–2020, with a large gap between the midstream and downstream regions and smaller differences between the upstream and midstream regions. The higher levels of the smart medical industry in downstream regions and large differences with other regions were mainly influenced by the advantages of geographic location, talent resources, and innovation capacity. Consequently, the middle and downstream regions, and the downstream regions, in particular, are limited by several aspects, such as resource endowment, location conditions, and innovation capacity, resulting in a lower absolute level of smart medical industry development, thus creating spatial differences between the same downstream regions.

Second, from the fluctuating status of the Gini coefficient between regions, the changes were highly consistent between upstream and downstream and between midstream and downstream, showing a trend of “down - up - down.” Thus, it can be seen that the level of variability among regions has been decreasing. The overall spatial non-equilibrium of the development of the smart medical industry in the Yangtze River Economic Zone was significant from the overall viewpoint, as shown in Table 6. For the evolution trend of the overall spatial variation of smart medical industry development in the Yangtze River Economic Zone, although the overall Gini coefficient increased slightly in 2018, it decreased in the following 2 years. Thus, the spatial imbalance was alleviated.

#### Identification of the sources of spatial unevenness in the development of the smart medical industry in the Yangtze River Economic Zone

According to the above calculations, the overall regional differences in the development of the smart medical industry in the Yangtze River Economic Zone are decreasing; however, variability still exists among regions, and the development status still presents unbalanced characteristics. To further explore the sources of the unbalanced characteristics of the spatial development of the smart medical industry, the Gini coefficient was decomposed based on the subgroup decomposition method. The results shown in Table 6 were obtained by quantitative analysis. The results showed that the uneven development of each region in the upper, middle, and lower reaches is the main reason for the unbalanced regional development of the smart medical industry. Its influence rate is 72.05% (2020) (Table 6). Meanwhile, the degree of smart medical industry development in each region also leads to the uneven development of the smart medical industry. However, the unevenness of development within a region has a low impact on the development differences between regions. Therefore, uneven development within regions is not a key factor because the smart medical industry is an innovative industry influenced by various aspects such as labor force quality, scientific and technological innovation capability, and economic capability. These influencing factors are unevenly distributed among each region, leading to the unbalanced spatial development of the smart medical industry in the Economic Zone of the Yangtze River.

## Conclusion and insights

### Research findings

Accurate identification of the dynamic and spatial characteristics of the development of the smart medical industry in the Yangtze River Economic Belt is an important prerequisite for the scientific formulation of policies to promote the development of the smart medical industry. This study used exploratory spatial data analysis to explore the dynamic spatial development and spatial imbalance of the smart medical industry in the Yangtze River Economic Belt. The findings of the study are discussed below.

First, between 2016 and 2020, the overall development of the smart medical industry in the Yangtze River Economic Zone showed fluctuations and an upward trend. While the level of smart medical industry development in the lower reaches of the Yangtze River was significantly higher than that in the middle and upper reaches, the level of industrial development in the middle and lower reaches of the river was still relatively low and displayed a large gap.

Second, the spatial correlation of the smart medical industry in the Yangtze River Economic Zone is significant and shows an upward trend in 2016–2020. The smart medical industry cluster pattern in the economic zone of the medical industry on the Yangtze River included mainly high-high, high-low, and low-low clusters; the high-high cluster was distributed primarily on the Yangtze River Delta, the high-low cluster was distributed in Sichuan, and Yunnan and Hunan were more dispersed. In addition, the smart medical industry in the Yangtze River Economic Zone also showed a multi-polar trend.

Third, the overall spatial differences in the development of the smart medical industry in the Yangtze River Economic Zone showed a trend of “fluctuating” and “narrowing.” The spatial imbalance of industrial development remained prominent, and the regional differences in the development of the upstream and downstream smart medical industry narrowed while the regional differences in the development of the smart medical industry in medium-sized industries expanded. The largest regional differences were in the upstream region from 2016 to 2020. Additionally, the level of development of the downstream smart medical industry is significantly higher than that of the upstream regions. However, the development gap was larger in the upstream region and narrowed in the middle and downstream regions. Finally, the contribution of regional differences is still 72.05% (2020), which is the main reason for the imbalance in the development space of the smart medical industry.

### Research insights

First, reducing the economic development gap and promoting synergistic regional development are necessary. Differential economic levels can easily lead to structural imbalances in the economic development of the Yangtze River Delta, disrupting the strategic planning of the Yangtze River Delta “community of destiny” and exacerbating the overall spatial differences in the development of the intelligent medical industry in the Yangtze River Economic Zone. First, we should strengthen the coordinated development of the three provinces and one city. Shanghai, as the economic highland of the Yangtze River Delta city cluster, should give full play to its leading edge in the Yangtze River Delta city cluster while enhancing the Yangtze River Delta “one map,” “one chess,” and “integration” “construction concepts,” appropriate to increase the economic depression tilt support so that they catch up with the Yangtze River Delta integration express, narrowing the economic gap within the Yangtze River Delta region. Second, we should strengthen the concept of building a community of shared interests between urban and rural areas. The spatial spillover effect of developing the smart medical industry in the Yangtze River Economic Belt should be fully addressed. The spatial autocorrelation of the development of the intelligent medical industry is significant, but there is still room to improve the local spatial autocorrelation. Therefore, relevant departments should understand the relationship between overall planning and local design, consider their own industrial development benefits, and contribute to the formation of new medical industry clusters. Moreover, the cities along the Yangtze River Economic Belt should clarify their own development direction and find the right direction to solve the problem of industrial isomorphism, strengthen the industrial agglomeration effect of favorable urban agglomeration, take into account the industrial agglomeration effect and regional agglomeration effect, eliminate the development obstacles of central cities, and promote the development of the intelligent medical industry in the Yangtze River Economic Belt. It is necessary to deal with the relationship between the whole and the local, improve the quality, and improve the efficiency and benefit of developing an intelligent medical industry.

Second, improving the regional industrial system and breaking the “homogeneous competition” is the key. Significant gradient differences in industrial structure and industrial “homogeneous competition” problems easily distort the industrial structure. It is difficult to give full play to the Yangtze River Delta's overall linkage effect. First, we should draw a map of the unified development of an industrial layout plan and the Yangtze River Delta intelligent medical industry. We should establish a coordination mechanism and an analysis and an evaluation mechanism for industrial layout planning in the region and have several independent third-party institutions effectively evaluate the layout of the wisdom medical industry proposed by the government. It is necessary to create a cross-regional wisdom medical industry cooperation demonstration base, deepen the strategic integration of industrial structure, develop a gradient layout of the industrial chain, and help wisdom medical enterprises to avoid the “misalignment competition” and win rapid development. The overall development of the smart medical industry in the Yangtze River Economic Zone should focus on both top-level design and increased factor support. Moreover, it is necessary to improve the institutional mechanism of government science and technology innovation, create a combination of standardized and decentralized intelligent medical industry development mechanisms, and accelerate the process of marketization, diversification, and intelligence, which include strengthening career development, actively introducing talents, increasing financial support, formulating policies and implementing programs from various aspects and angles, promoting deep integration and positive interaction between the intelligent medical industry and other strategic emerging industries in the Yangtze River Economic Zone, and developing new paths as well as the development and utilization of resources and equipment based on achieving rational innovation.

Giving full play to the role of government coordination is vital. In promoting the integrated development of the Yangtze River Delta region's intelligent medical industry, the roles of “strong government” and “strong market” should be played, but in different positions. The “strong government” should be reflected in two aspects: First, the implementation of competition law is the core so as to create a good environment to give full play to the role of market competition, such as the suppression of monopolies to encourage competition and to regulate the behavior of market players; the other is to carry on the regional union oriented by the public social goal and join hands to do some things that the market fails to do, such as regional infrastructure construction and soft environment optimization. Relevant departments should understand the relationship between overall planning and local design, consider their own industrial development benefits, and contribute to the formation of new medical industry clusters. Secondly, cities along the Yangtze River Economic Belt should clarify their own development direction and find their own development direction to solve the problem of industrial isomorphism. We should strengthen the industrial agglomeration effect of favorable city clusters, take into account the industrial agglomeration effect and regional agglomeration effect, eliminate obstacles to the development of central cities, promote the Yangtze River Economic Belt intelligent medical industry development, deal with the relationship between the whole and local, improve the quality, and improve the efficiency and effectiveness of the development of the intelligent medical industry.

## Data availability statement

The original contributions presented in the study are included in the article/supplementary material, further inquiries can be directed to the corresponding author.

## Author contributions

XuZ and JX contributed to the analysis and interpretation of data for the study and wrote the first draft of the manuscript. LY designed the framework for this study. XiZ contributed to the acquisition of data for this study. All authors approved the final manuscript.
